# Influence of control selection in genome-wide association studies: the example of diabetes in the Framingham Heart Study

**DOI:** 10.1186/1753-6561-3-s7-s113

**Published:** 2009-12-15

**Authors:** Delphine D Fradin, M Daniele Fallin

**Affiliations:** 1 Department of Epidemiology, Johns Hopkins Bloomberg School of Public Health, 615 North Wolfe Street, Baltimore, Maryland 21205, USA

## Abstract

Epidemiologic study designs represent a major challenge for genome-wide association studies. Most such studies to date have selected controls from the pool of participants without the disease of interest at the end of the study time. These choices can lead to biased estimates of exposure effects. Using data from the Framingham Heart Study (Genetic Analysis Workshop 16 Problem 2), we evaluate the impact on genetic association estimates for designs with control selection based on status at the end of a study (case exclusion (CE) sampling) to control selection based on incidence density (ID) sampling, when controls are selected from the pool of participants who are disease-free at the time a case is diagnosed. Cases are defined as those diagnosed with type 2 diabetes (T2D). We estimated odds ratios for 18 previously confirmed T2D variants using 189 cases selected by ID sampling and using 231 cases selected by CE sampling. We found none of these single-nucleotide polymorphisms to be significantly associated with T2D using either design. Because these empirical analyses were based on a small number of cases and on single-nucleotide polymorphisms with likely small effect sizes, we supplemented this work with simulated data sets of 500 cases from each strategies across a variety of allele frequencies and effect sizes. In our simulated datasets, we show ID sampling to be less biased than CE, although CE shows apparent increased power due to the upward bias of point estimates. We conclude that ID sampling is an appropriate option for genome-wide association studies.

## Background

The genetic architecture of type 2 diabetes (T2D) appears to be composed of several genes, each of which has a modest impact on disease risk. Despite significant advances in our understanding of the genetic determinants of the monogenic forms of diabetes, the definitive identification of genes that increase risk of common T2D in the general population has been far more elusive. However, a string of recent genome-wide association studies (GWAS) has given promising clues to additional genes involved in common T2D risk.

GWAS offer an approach to gene discovery unbiased with regard to presumed functions or locations in the genome. The common method of control selection used for many GWAS is to form a single pool of potential controls consisting of subjects who were not cases by the end of the study period. However, this method has been shown by Greenland and Thomas [1] and Lubin and Gail [2] to lead to biased estimates of the rate ratio. This bias has been termed "case-exclusion bias". Moreover, differences in the origin of populations of cases and controls can arise if the two groups are recruited independently or have different inclusion criteria, and the presence of population stratification can lead to greater than nominal type I error rate.

Another method of control selection, termed "incidence density sampling", uses subjects who survived to the time of case occurrence to make a pool of potential controls for each case. The pool of potential controls may include subjects who later become cases and subjects who develop other diseases. This nested case-control design can be a very efficient approach to obtain unbiased estimates of relative risks associated with genetic variants.

In this study, we use the GWAS data from the Framingham Heart Study (FHS, Genetic Analysis Workshop 16 Problem 2) to compare the influence of control selection on the results for T2D.

## Methods

### FHS

The FHS is a community-based, multigenerational, longitudinal study of cardiovascular disease and its risk factors, including diabetes. The FHS began in 1948 to investigate the causes of heart disease. Men and women between the ages of 28 and 62 years were recruited and followed prospectively over time. Beginning in 1971, offspring of the Original Cohort were recruited as part of the Framingham Offspring Study. There are a total of 6752 subjects. There are 765 pedigrees with 2 to 301 genotyped subjects: 134 pedigrees with 2, 123 with 3, 98 with 4, 85 with 5, 177 with 6 to 10, 72 with 11 to 15, 30 with 16 to 20, and 46 with more than 20.

### Genotyping

FHS GWAS data were generated on the Affymetrix 250 k Sty, 250 k Nsp, and the supplemental 50 k platforms. Single-nucleotide polymorphisms (SNPs) were selected for analyses were based on previously reported GWAS [3,4]. The genes and SNPs used in this study to represent the 18 most significant T2D SNPs from these GWAS are: peroxisome proliferator-activated receptor gamma (*PPARG*; rs1801282); insulin-like growth factor two binding protein 2 (*IGF2BP2*; rs4402960); cyclin-dependent kinase 5, a regulatory subunit-associated protein1-like 1 (*CDKAL1*; rs7754840 and rs10946398); a variant found near cyclin-dependent kinase inhibitor 2A/2B (*CDKN2A/2B*; rs10811661 and rs564398); hematopoietically expressed homeobox (*HHEX*; rs5015480 (*r*^2 ^= 1 with rs1111875); transcription factor-7-like 2 (*TCF7L2*; rs10885409 and rs7901695 (*r*^2 ^= 0.8 with rs7903146)); potassium inwardly rectifying channel subfamily J member 11(*KCNJ11*; rs5215 (*r*^2 ^= 0.89 with rs5219)); fat mass obesity-associated gene (*FTO*; rs9939609 and rs8050136); tetraspanin 8/leucine-rich repeat-containing G protein-coupled receptor 5 (*TSPAN8/LGR5*; rs7961581); cell division cycle 123 (*CDC123*; rs4747969 (*r*^2 ^= 0.83 with rs12779790)); Wolfram syndrome 1 (*WFS1*; rs4689394 (*r*^2 ^= 1 with rs10010131)); ADAM metallopeptidase with thrombospondin type 1 motif, 9 (*ADAMTS9*; rs4607103); thyroid adenoma associated (*THADA*; rs13431070 (*r*^2 ^= 1 with rs7578597); and JAZF zinc finger 1 (*JAZF1*; rs864745).

### Case-control definitions

Cases were defined as people with a diagnosis of type 2 diabetes (T2D) during follow-up of the FHS cohort. Cases were born during the first, the second, or the third generation of the FHS. The age at diagnosis for 231 unrelated male and female cases was 20 to 80 years old.

In our nested incidence density case-control approach, 10 individually matched controls were selected with replacement from members of the cohort who did not have a T2D diagnosis at the time when the case was identified. Age is a strong risk factor for T2D disease, and so controls were always selected among participants of the same age at enrollment as the cases ( ± 5 years). Controls were additionally matched on sex and body mass index (BMI) at enrollment ( ± 2 kg/m^2^). For every case, ten randomly chosen controls were selected by incidence density sampling. Cases and controls were not members of the same family. In our case-exclusion approach, controls were selected as members of the FHS who never received a T2D diagnosis during any of the recorded follow-up. We then adjusted for age, sex, and BMI matching criteria as in our nested case-control approach.

### Statistical analyses

As a quality control measure, we tested for Hardy-Weinberg disequilibrium in controls using an exact test. All markers are in Hardy-Weinberg equilibrium in the observed FHS data and in all simulated samples. All individuals had complete data for sex, age, BMI, and diabetes except 15 controls in the incidence density (ID) sample and 28 in the case exclusion (CE) sample for whom BMI at enrollment was not available. All SNPs had no more than 10.4% missing data, which we judged to be acceptable.

Genetic associations with T2D (odds ratios, confidence intervals, and statistical tests) were estimated and tested using a conditional logistic regression under the additive model for the ID sampling approach and using logistic regression, adjusted for matching variables, in the case-exclusion approach. These analyses were carried out in SAS software using the PHREG procedure.

### Simulations

Simulations were used to investigate control selection effects in a larger sample of individuals than that in the observed FHS data, and with SNPs having higher effect sizes. We simulated 11 sets of 100 replicates according to varying minor allele frequencies and generating hazard ratios. These simulations were used to estimate bias and power between the control sampling designs. A SAS program was used to simulate diabetes as a function of SNP genotype. We generated data sets of 10,000 individuals with SNP genotypes assigned probabilistically according to allele frequencies of 0.10, 0.30, or 0.50. We then assigned diabetes status and time of onset using an exponential model based on SNP hazard ratios from 1.3 to 3 (see Table 1). We selected five controls for each case according to the ID (risk set) sampling scheme and set a 5:1 control:case ratio for the CE sampling at end of follow up. We then estimated odds ratios (ORs), confidence intervals (CIs) and performed tests of association for each SNP. We repeated this 100 times to report average bias and estimated power for each SNP (defined as the proportion with statistically significant association (*p *< 0.05)). Bias ratios between ID and CE methods were estimated by the ratio "calculated OR per method/generating hazard ratio in simulations".

**Table 1:** OR and bias from simulated cohorts under different control sampling designs based on 100 replicates for each design

	Generated hazard ratio		ID sampling			CE sampling		
			
SNP	OR	MAF	OR (CI)^a^	Bias ratio	Power	OR (CI)	Bias ratio	Power
Gene1.3_0.1	1.3	0.1	1.29 (0.83-1.99)	0.99	0.43	1.38 (0.86-2.18)	1.06	0.66
Gene1.3_0.3	1.3	0.3	1.30 (0.79-1.26)	1.00	0.97	1.37 (0.77-1.29)	1.04	0.98
Gene1.3_0.5	1.3	0.5	1.29 (0.80-1.25)	0.99	0.96	1.36 (0.78-1.29)	1.04	0.99
Gene1.5_0.1	1.5	0.1	1.57 (0.49-2.05)	1.04	0.92	1.59 (0.52-1.90)	1.06	0.91
Gene1.5_0.3	1.5	0.3	1.54 (0.58-1.72)	1.02	1.00	1.61 (0.59-1.71)	1.08	1.00
Gene1.5_0.5	1.5	0.5	1.55 (0.55-1.82)	1.03	1.00	1.62 (0.57-1.76)	1.09	1.00
Gene1.8_0.1	1.8	0.1	1.90 (0.27-3.47)	1.06	1.00	2.03 (0.24-4.18)	1.13	1.00
Gene1.8_0.3	1.8	0.3	1.75 (0.38-2.64)	0.97	1.00	1.96 (0.30-3.31)	1.09	1.00
Gene1.8_0.5	1.8	0.5	1.77 (0.36-2.80)	0.99	1.00	1.97 (0.29-3.47)	1.09	1.00
Gene2_0.5	2.0	0.5	1.78 (0.52-2.08)	0.89	1.00	2.64 (0.25-3.10)	1.32	1.00
Gene3_0.5	3.0	0.5	2.92 (0.47-3.44)	0.97	1.00	3.67 (0.38-4.34)	1.22	1.00

^a^CI, 95% confidence interval

## Results and discussion

In order to maximize precision, we chose a ratio of 10 controls per case for both sampling strategies in the FHS data. Because we did not have exact dates and BMI at onset of diabetes, we used the age at enrollment, i.e., the age at Visit 1, and BMI at enrollment to match cases and controls. To accommodate the effect of random ID control selection, we repeated random sampling and conditional logistic regression 10 times. The distribution of OR estimates obtained in each analysis showed wide variability across replicates, with a coefficient of variation from 14% to 20% per SNP among the ID sampling replicates. We report the average OR from these 10 replicates in Table 2, along with confidence limits based on the method of Rubin [5] that takes within-replicate and across-replicate variation into account. We also show the average *p*-value per SNP to indicate whether statistical significance was achieved in any replicate.

**Table 2:** OR for T2D SNPs from previous meta-analysis and in the FHS data under different control sampling designs

	Previous studies		ID sampling	CE sampling
			
SNP	OR (CI)	MAF	OR (CI)^a^	OR (CI)
rs4607103	1.09 (1.06-1.12)	0.20	1.26 (0.56-2.83)	1.28 (0.71-2.35)
rs4747969	1.11 (1.07-1.14)	0.27	1.21 (0.53-2.74)	1.21 (0.63-2.29)
rs10946398	1.14 (1.11-1.17)	0.31	1.26 (0.85-1.86)	1.24 (0.73-2.09)
rs7754840	1.12 (1.08-1.16)	0.31	1.24 (0.84-1.85)	1.30 (0.75-2.27)
rs10811661	1.20 (1.14-1.25)	0.21	1.32 (0.66-2.63)	1.14 (0.57-2.28)
rs564398	1.12 (1.07-1.17)	0.37	0.91 (0.63-1.31)	1.21 (0.73-2.01)
rs8050136	1.17 (1.12-1.22)	0.45	1.05 (0.73-1.48)	1.69 (1.05-2.72)
rs9939609	1.11 (1.02-1.20)	0.45	1.16 (0.74-1.47)	1.69 (1.06-2.73)
rs5015480	1.15 (1.10-1.19)	0.43	1.25 (0.81-1.93)	1.21 (0.72-2.03)
rs4402960	1.14 (1.11-1.18)	0.29	1.01 (0.68-1.50)	1.02 (0.61-1.70)
rs864745	1.10 (1.07-1.13)	0.48	1.24 (0.80-1.91)	1.38 (0.83-2.29)
rs5215	1.14 (1.10-1.19)	0.41	1.14 (0.70-1.84)	1.03 (0.62-1.70)
rs1801282	1.14 (1.08-1.20)	0.07	1.02 (0.61-1.69)	1.01 (0.45-2.93)
rs10885409	1.29 (1.07-1.54)	0.42	1.25 (0.81-1.92)	1.50 (0.77-2.93)
rs7901695	1.37 (1.31-1.43)	0.28	1.34 (0.85-2.09)	1.07 (0.53-2.16)
rs13431070	1.15 (1.10-1.20)	0.08	1.33 (0.58-3.05)	1.02 (0.46-2.29)
rs7961581	1.09 (1.06-1.12)	0.23	1.16 (0.84-1.61)	1.03 (0.60-1.75)
rs4689394	1.11 (1.08-1.16)	0.27	1.18 (0.71-1.95)	1.23 (0.74-2.04)

^a^Average OR and CI (95% confidence interval) across 10 replicates

We failed to find any significant association with any of the 18 previously reported SNPs using ID sampling or CE sampling in FHS (Table 2). We included 18 SNPs with convincing association evidence; however, two important SNPs were missing in our genotyping data (rs757210 in *TCF2 *and rs13266634 in *SLC30A8*), and could not be considered in the FHS. One drawback of our study is the limited number of T2D cases, despite the very large database. With only 189 incident cases and 231 total cases, our study had low power to detect genetic association between SNPs and T2D, especially considering the expected magnitudes of association based on previous reports. Owing to the large CIs of the ORs in our two scenarios, the results would have been less conclusive than those of the previous studies conducted in larger sample (>1000 cases). An alternative explanation for the low power is that we considered each SNP separately rather than a combination of variants acting additively on risk, which may have a large effect.

Because the empirical data are hard to interpret due to the small number of cases and small effect sizes, we further addressed differences between control sampling methods via simulation with higher sample sizes and effect sizes. For each simulating scenario, we simulated 100 cohort data sets, each with approximately 500 cases, as described in the Methods section (Table 1). These simulations show that when more precision can be obtained and higher effect sizes are considered, ID sampling does indeed have less bias, while CE methods have a slight upward bias, leading to the appearance of increase power. We suggest that this increased power should be considered with caution given the bias, and recommend ID sampling as the appropriate strategy for case-control analyses nested in cohorts.

## List of abbreviations used

BMI: Body mass index; CE: Case exclusion; CI: Confidence interval; FHS: Framingham Heart Study; GWAS: Genome-wide association studies; ID: Incidence density; OR: Odds ratio; SNP: Single-nucleotide polymorphism; T2D: Type 2 diabetes.

## Competing interests

The authors declare that they have no competing interests.

## Authors' contributions

DDF carried out analyses and drafted the manuscript. MDF aided in the design of the analysis and provided intellectual input for the discussion. Both authors read and approved the final manuscript.

## References

[B1] Greenland S Thomas DC On the need for the rare disease assumption in case-control studies Am J Epidemiol 1982 116 547 553 7124721 10.1093/oxfordjournals.aje.a113439

[B2] Lubin JH Gail MH Biased selection of controls for case-control analyses of cohort studies Biometrics 1984 40 63 75 10.2307/2530744 6375751

[B3] Frayling TM Genome-wide association studies provide new insights into type 2 diabetes aetiology Nat Rev Genet 2007 8 657 662 10.1038/nrg2178 17703236

[B4] Lango H UK Type 2 Diabetes Genetics Consortium Palmer CN Morris AD Zeggini E Hattersley AT McCarthy MI Frayling TM Weedon MN Assessing the combined impact of 18 common genetic variants of modest effect sizes on type 2 diabetes risk Diabetes 2008 57 3129 3135 18591388 10.2337/db08-0504 PMC2570411

[B5] Rubin W Multiple Imputation for Nonresponse in Surveys 1987 New York, Wiley

